# Supplementary material for: A transcription factor-sRNA-mediated double-negative feedback loop confers pathogen-specific control of quorum-sensing genes
Source: mBio. 2026 May 11;17(6):e00633-26. doi: 10.1128/mbio.00633-26 (PMC13251380; doi:10.1128/mbio.00633-26)
Supplement: Supplemental tables — Tables S1 to S3. [file mbio.00633-26-s0003.pdf]

Title: Supplementary Table S1 - Strain List
Description: This table contains strains used in this study.

**Supplementary Table 1**		
**Strain**	**Source**	**Identifier**
C6706; Δ*tdh*; *vqmA*-$_{FLAG}$; *lacZ*::P*vqmR-lux*; *vc1807*::Cm^R	This work	KD4
C6706; Δ*tdh*; $vqmA_{C134A\text{-}FLAG}$; *lacZ*::P*vqmR-lux*; *vc1807*::Cm^R	This work	KD37
C6706; Δ*tdh*; Δ*vc0122*; $vqmA_{C134A\text{-}FLAG}$; *lacZ*::P*vqmR-lux*; *vc1807*::Kan^R	This work	KD64
C6706; Δ*tdh*; Δ*vc2614*; $vqmA_{C134A\text{-}FLAG}$; *lacZ*::P*vqmR-lux*; *vc1807*::Kan^R	This work	KD88
C6706; Δ*tdh*; Δ*vca0566*; $vqmA_{C134A\text{-}FLAG}$; *lacZ*::P*vqmR-lux*; *vc1807*::Kan^R	This work	KD85
C6706; Δ*tdh*; Δ*luxT*; $vqmA_{C134A\text{-}FLAG}$; *lacZ*::P*vqmR-lux*; *vc1807*::Cm^R	This work	KD38
C6706; Δ*tdh*; Δ*vc0165*; $vqmA_{C134A\text{-}FLAG}$; *lacZ*::P*vqmR-lux*; *vc1807*::Cm^R	This work	KD40
C6706; Δ*tdh*; Δ*vc0433*; $vqmA_{C134A\text{-}FLAG}$; *lacZ*::P*vqmR-lux*; *vc1807*::Cm^R	This work	KD46
C6706; Δ*tdh*; Δ*vc0595*; $vqmA_{C134A\text{-}FLAG}$; *lacZ*::P*vqmR-lux*; *vc1807*::Cm^R	This work	KD41
C6706; Δ*tdh*; Δ*vc0773*; $vqmA_{C134A\text{-}FLAG}$; *lacZ*::P*vqmR-lux*; *vc1807*::Cm^R	This work	KD44
C6706; Δ*tdh*; Δ*vc0833*; $vqmA_{C134A\text{-}FLAG}$; *lacZ*::P*vqmR-lux*; *vc1807*::Cm^R	This work	KD35
C6706; Δ*tdh*; Δ*vc0907*; $vqmA_{C134A\text{-}FLAG}$; *lacZ*::P*vqmR-lux*; *vc1807*::Kan^R	This work	KD99
C6706; Δ*tdh*; Δ*vc1141*; $vqmA_{C134A\text{-}FLAG}$; *lacZ*::P*vqmR-lux*; *vc1807*::Cm^R	This work	KD43
C6706; Δ*tdh*; Δ*vca0707*; $vqmA_{C134A\text{-}FLAG}$; *lacZ*::P*vqmR-lux*; *vc1807*::Cm^R	This work	KD36
C6706; Δ*tdh*; Δ*vca0708*; $vqmA_{C134A\text{-}FLAG}$; *lacZ*::P*vqmR-lux*; *vc1807*::Cm^R	This work	KD42
C6706; Δ*tdh*; Δ*vca0965*; $vqmA_{C134A\text{-}FLAG}$; *lacZ*::P*vqmR-lux*; *vc1807*::Cm^R	This work	KD39
C6706; Δ*tdh*; Δ*vqmA*; *lacZ*::P*vqmR-lux*; *vc1807*::P_{BAD}-*vqmA*-$Spec^R$	This work	KD12
C6706; Δ*tdh*; Δ*vqmA*; Δ*vc0122*; *lacZ*::P*vqmR-lux*; *vc1807*::P_{BAD}-*vqmA*-$Spec^R$	This work	KD80
C6706; Δ*tdh*; Δ*vqmA*; Δ*vc2614*; *lacZ*::P*vqmR-lux*; *vc1807*::P_{BAD}-*vqmA*-$Spec^R$	This work	KD113
C6706; Δ*tdh*; Δ*vqmA*; Δ*vca0566*; *lacZ*::P*vqmR-lux*; *vc1807*::P_{BAD}-*vqmA*-$Spec^R$	This work	KD57
C6706; Δ*tdh*; Δ*vqmA*; Δ*luxT*; *lacZ*::P*vqmR-lux*; *vc1807*::P_{BAD}-*vqmA*-$Spec^R$	This work	KD13
C6706; Δ*tdh*; Δ*luxT*; *vqmA*-$_{FLAG}$; *lacZ*::P*vqmR-lux*; *vc1807*::Kan^R	This work	KD9
C6706; Δ*tdh*; Δ*luxT*; *vqmA*-$_{FLAG}$; *lacZ*::P*vqmR-lux*; *vc1807*::Cm^R	This work	KD50
C6706; Δ*tdh*; Δ*hapR*; *vqmA*-$_{FLAG}$; *vc1807*::$Spec^R$	This work	KD106
C6706; Δ*tdh*; Δ*hapR*; Δ*luxT*; *vqmA*-$_{FLAG}$; *vc1807*::$Spec^R$	This work	KD157
C6706; Δ*tdh*; Δ*hapR*; Δ*vqmA*; *vc1807*::$Spec^R$	This work	KD108
C6706; Δ*tdh*; Δ*hapR*; Δ*vqmA*; Δ*luxT*; *vc1807*::$Spec^R$	This work	KD104
Saccharomyces cerevisiae YF2	Bassler Lab Collection	AAM25
Escherichia coli S17	Bassler Lab Collection	
Escherichia coli BL21	Bassler Lab Collection	

Title: Supplementary Table S2 - Plasmid List
Description: This table lists plasmids used in this study.

Supplementary Table 2		
**Plasmids**	**Source**	**Identifier**
pEVS-P_{BAD}	Bassler Lab Collection	pEVS-P_{BAD}
pEVS-P_{BAD}-*luxT*$_{Vc\text{-}FLAG}$	This work	pKD225
pET-15b-His6X-*luxT*$_{Vc}$	This work	pKD01
pET-15b-His6X-*luxT*$_{Vc\ \Delta 8\text{-}AA}$	This work	pKD193
pRE112-*vca0122*	This work	pKD56
pRE112-*vc2614*	This work	pKD58
pRE112-*vca0566*	This work	pKD48
pEVS-P_{BAD}-$_{FLAG}$-*Spec*^{R}	This work	pJSV1081
pEVS-P_{BAD}-*luxT*-$_{FLAG}$-*Spec*^{R}	This work	pJSV1082
pEVS-P_{BAD}-*wigR*-$_{FLAG}$-*Spec*^{R}	This work	pJSV1084
pEVS-P_{BAD}-*crp*-$_{FLAG}$-*Spec*^{R}	This work	pJSV1086
pEVS-P_{BAD}-*acy*-$_{FLAG}$-*Spec*^{R}	This work	pJSV1087

Title: Supplementary Table S3 - Primer List
Description: This table lists sequences used for cloning or for EMSAs in this study.

Supplementary Table 3		
**Name**	**Sequence**	**Purpose**
vca1078 Mugent F	ATTGAAATGCGTCTGTCGCAAATCAAACAGCG	Strain construction
vca1078 Mugent R	AAACACCGCAATCATCCCTGCGACTAG	Strain construction
vc1807 Mugent F	TTTAAAGGGGATCAGTGACCG	Strain construction
vc1807 Mugent R	CAATTTTGCTTTTGGACCATCCC	Strain construction
luxT 3k up F	GCAGCGCAACAGTAGCAAGCAGAAGTAC	Strain construction
luxT 3k down R	TGCTCAGCATTAACGGTAATGCTGCTCGC	Strain construction
YCC *vc0122* up F	CTACATATTCATCGTTCATTGTAATTAATGATGAAATCCTAGTTGGTGGGCGAGAGTG	Strain construction
YCC *vc0122* up R	CACTCTCGCCCACCAACTAGGATTTCATCATTAATTACAATGAACGATGAATATGTAG	Strain construction
vc0122 pRE down F	CCGGAATTGATCCGCCGTCTGGCGGATCGGGGATCGGGCCCTATCACTTATTCAGGCG	Strain construction
vc0122 pRE down R	CGCCTGAATAAGTGATAGGGCCCGATCCCCGATCCGCCAGACGGCGGATCAATTCCGG	Strain construction
YCC *vc2614* up F	CTACATATTCATCGTTCATTGTAATTAATTTAGCCGCCTGCTCACAACGTATCGAACA	Strain construction
YCC *vc2614* up R	TGTTCGATACGTTGTGAGCAGGCGGCTAAATTAATTACAATGAACGATGAATATGTAG	Strain construction
vc2614 pRE down F	CAGAGCTGGCACAATATGCCCATTGGGCAGGGATCGGGCCCTATCACTTATTCAGGCG	Strain construction

vc2614 pRE down R	CGCCTGAATAAGTGATAGGGCCCGATCCCTGCCCAATGGGCATATTGTGCCAGCTCTG	Strain construction
YCC *wigR* up F	CTACATATTCATCGTTCATTGTAATTAATGTACGATTTGGTATTCACCCAGTCGCTGG	Strain construction
YCC *wigR* up R	CCAGCGACTGGGTGAATACCAAATCGTACATTAATTACAATGAACGATGAATATGTAG	Strain construction
wigR down pRE F	TTGAGGGCAGGGTACAACAAAAACCTCGGGGATCGGGCCCTATCACTTATTCAGGCGT	Strain construction
wigR down pRE R	ACGCCTGAATAAGTGATAGGGCCCGATCCCCGAGGTTTTTGTTGTACCCTGCCCTCAA	Strain construction
pEVS P_{BAD}-*luxT* F	CTGTTTCTCCGGATCCAAGGAGTGTATTCTTGACGTTAGAAAAGAGCCTGACCATGCC	Plasmid construction
pEVS P_{BAD}-*luxT* R	GGCATGGTCAGGCTCTTTTCTAACGTCAAGAATACACTCCTTGGATCCGGAGAAACAG	Plasmid construction
luxT-$_{FLAG}$ pEVS F	ATATCGACTACAAAGATGACGATAAATAGTTCTTCACCTTCTGCCTACTGGATCCGGT	Plasmid construction
luxT-$_{FLAG}$ pEVS R	ACCGGATCCAGTAGGCAGAAGGTGAAGAACTATTTATCGTCATCTTTGTAGTCGATAT	Plasmid construction
luxT-$_{FLAG}$ F	TGGGGCACTCATTAGTCAGCATGGTGAATGACTACAAAGACCATGACGGTGATTATAA	Plasmid construction
luxT-$_{FLAG}$ R	TTATAATCACCGTCATGGTCTTTGTAGTCATTCACCATGCTGACTAATGAGTGCCCCA	Plasmid construction
P*vqmR*$_{Vc}$ WT LuxT site	TTATGTCGGTTTCCGAGTATTGCGCGAGCTGTAATGTTGACTCAAACAATTATGCATAAAGGGGGGATTTCCCCCCTTTTTCATTTGTACCGCGTTCGGTAAAGTACAAACCAGAGCATGAGTTGCATGACTGATGCTTGGTATCAATATGATACCTCTG	EMSA probe
P*vqmR*$_{Vc}$ Scrambled LuxT site	TTATGTCGGTTTCCGAGTATTGCGCGAGCTGTAATGTTGACTCAAACAATTATGCATAAAGGGGGGATTTCCCCCCTTTTTCAATCTACGATGCGTATGCGTTGTACAAACCAGAGCATGAGTTGCATGACTGATGCTTGGTATCAATATGATACCTCTG	EMSA probe
P*vqmR*$_{Vh}$ LuxT site	CGTTATTGGCTTGTTGCTTCGCGTATTCAAGAGGATCTTTGTTGACTCAAACAATTATGCATAAAGGGGGGATTTCCCCCCTTTTTCATATGCTCACAATTCAATACAGTACAAACCGAAATGACTAAAAAAATTTCATTCATTATTAACGGCCAGGTTGAAATCTTTCCCGAAAGTGATGATTTAGTGCAATCCATTAA	EMSA probe
P*vqmR*$_{Vs}$ LuxT site	CGTTATTGGCTTGTTGCTTCGCGTATTCAAGAGGATCTTTGTTGACTCAAACAATTATGCATAAAGGGGGGATTTCCCCCCTTTTTCATAAGCATTCCTAGGAGTAGAGTACAAACCGAAATGACTAAAAAAATTTCATTCATTATTAACGGCCAGGTTGAAATCTTTCCCGAAAGTGATGATTTAGTGCAATCCATTAA	EMSA probe
P*vqmR*$_{Vm}$ LuxT site	CGTTATTGGCTTGTTGCTTCGCGTATTCAAGAGGATCTTTGTTGACTCAAACAATTATGCATAAAGGGGGGATTTCCCCCCTTTTTCATTTGTATCCTGTTCGGTAAAGTACAAACCGAAATGACTAAAAAAATTTCATTCATTATTAACGGCCAGGTTGAAATCTTTCCCGAAAGTGATGATTTAGTGCAATCCATTAA	EMSA probe
pEVS-*luxT*$_{8AAKO}$ F	CTGTTTCTCCGGATCCAAGGAGTGTATTCATGCCTAAGCGTAGTAAAGAAGATACTGA	Plasmid construction
pEVS-*luxT*$_{8AAKO}$ R	TCAGTATCTTCTTTACTACGCTTAGGCATGAATACACTCCTTGGATCCGGAGAAACAG	Plasmid construction
pEVS-*luxT*$_{Vh}$ F	CTGTTTCTCCGGATCCAAGGAGTGTATTCATGCCAAAGCGTAGTAAAGAAGATACCGA	Plasmid construction
pEVS-*luxT*$_{Vh}$ R	TCGGTATCTTCTTTACTACGCTTTGGCATGAATACACTCCTTGGATCCGGAGAAACA	Plasmid construction
luxT$_{Vh\text{-}FLAG}$ F	GTCGTTCGTTAATTCAAATGAGCAAATAAGACTACAAAGACCATGACGGTGATTATAA	Plasmid construction
lux$_{Vh\text{-}FLAG}$ R	TTATAATCACCGTCATGGTCTTTGTAGTCTTATTTGCTCATTTGAATTAACGAACGAC	Plasmid construction
P*swrZ*$_{Vh\ -110\ to\ +20}$	TCAGCCCTCTTTTTTTATATATTTAATCTTGCCCTCATAATCAGTACGCTGTATTATCAATTACAACCGAACTACGTACTGTTTAGTTGTCGTCACGATAATAAAATTTCATGAGGTTGCTTGTGTCTAG	EMSA probe
Motif 1-like LuxT site scrambled P*swrZ*$_{Vh\ -110\ to\ +20}$	TCAGCCCTCTTTTTTTATATATTTAATCTTGCCCTCATAATCAGTACGCTGTATTATCAATTACAACCGAACTACGTACTGTGATCTTAATGAGAGTAATCTACTATTTCATGAGGTTGCTTGTGTCTAG	EMSA probe
P*swrZ*$_{Vh\ -110\ to\ -81}$ F	TCAGCCCTCTTTTTTTATATATTTAATCTT	EMSA probe
P*swrZ*$_{Vh\ -80\ to\ -56}$ F	GCCCTCATAATCAGTACGCTGTATT	EMSA probe
P*swrZ*$_{Vh\ -80\ to\ -56}$ R	AATACAGCGTACTGATTATGAGGGC	EMSA probe
P*swrZ*$_{Vh\ -55\ to\ -31}$ F	ATCAATTACAACCGAACTACGTACT	EMSA probe
P*swrZ*$_{Vh\ -55\ to\ -31}$ R	AGTACGTAGTTCGGTTGTAATTGAT	EMSA probe

P*swrZ*$_{Vh\ -30\ to\ -6}$ F	GTTTAGTTGTCGTCACGATAATAAA	EMSA probe
P*swrZ*$_{Vh\ -30\ to\ -6}$ R	TTTATTATCGTGACGACAACTAAAC	EMSA probe
P*swrZ*$_{Vh\ -5\ to\ 20}$ R	CTAGACACAAGCAACCTCATGAAAT	EMSA probe
P*swrZ*$_{Vh\ -40\ to\ -6}$	ACTACGTACTGTTTAGTTGTCGTCACGATAATAAA	EMSA probe
P*swrZ*$_{Vh\ -75\ to\ -51}$	CATAATCAGTACGCTGTATTATCAA	EMSA probe
P*swrZ*$_{Vh\ -60\ to\ -31}$	GTATTATCAATTACAACCGAACTACGTACT	EMSA probe
P*swrZ*$_{Vh\ -80\ to\ -61}$	GCCCTCATAATCAGTACGCT	EMSA probe
Scrambled P*swrZ*$_{Vh\ -75\ to\ -51}$	ATCATGTACTACATACGTATTGAAC	EMSA probe
motif 2L F	TAATCAGTACGCTGT	EMSA probe
motif 2R F	AACTACGTACTGTTT	EMSA probe
motif 2L scr F	GCGTATGATATTCAC	EMSA probe
motif 2R scr F	TGCATTTATCACATG	EMSA probe
motif 2L R	ACAGCGTACTGATTA	EMSA probe
motif 2R R	AAACAGTACGTAGTT	EMSA probe
motif 2L scr R	GTGAATATCATACGC	EMSA probe
motif 2R scr R	CATGTGATAAATGCA	EMSA probe
Motif 2L LuxT site scrambled P*swrZ*$_{Vh\ -110\ to\ +20}$ Vh	TCAGCCCTCTTTTTTTATATATTTAATCTTGCCCTCAGCGTATGATATTCACATTAT CAATTACAACCGAACTACGTACTGTTTAGTTGTCGTCACGATAATAAAATTTCATG AGGTTGCTTGTGTCTAGTCCTACCCTTACCGACAAAGTTTC	EMSA probe
Motif 2R LuxT site scrambled P*swrZ*$_{Vh\ -110\ to\ +20}$	TCAGCCCTCTTTTTTTATATATTTAATCTTGCCCTCATAATCAGTACGCTGTATTAT CAATTACAACCGTGCATTTATCACATGAGTTGTCGTCACGATAATAAAATTTCATG AGGTTGCTTGTGTCTAGTCCTACCCTTACCGACAAAGTTTC	EMSA probe
Motif 2L and 2R LuxT site scrambled P*swrZ*$_{Vh\ -110\ to\ +20}$	TCAGCCCTCTTTTTTTATATATTTAATCTTGCCCTCAGCGTATGATATTCACATTAT CAATTACAACCGTGCATTTATCACATGAGTTGTCGTCACGATAATAAAATTTCATG AGGTTGCTTGTGTCTAGTCCTACCCTTTACCGACAAAGTTTC	EMSA probe
